# Seeds and Seedlings in a Changing World: A Systematic Review and Meta-Analysis from High Altitude and High Latitude Ecosystems

**DOI:** 10.3390/plants10040768

**Published:** 2021-04-14

**Authors:** Jerónimo Vázquez-Ramírez, Susanna E. Venn

**Affiliations:** Centre for Integrative Ecology, School of Life and Environmental Sciences, Deakin University, 221 Burwood Hwy, Burwood, VIC 3125, Australia; susanna.venn@deakin.edu.au

**Keywords:** alpine, tundra, treeline, climate change, germination, seedling establishment

## Abstract

The early life-history stages of plants, such as germination and seedling establishment, depend on favorable environmental conditions. Changes in the environment at high altitude and high latitude regions, as a consequence of climate change, will significantly affect these life stages and may have profound effects on species recruitment and survival. Here, we synthesize the current knowledge of climate change effects on treeline, tundra, and alpine plants’ early life-history stages. We systematically searched the available literature on this subject up until February 2020 and recovered 835 potential articles that matched our search terms. From these, we found 39 studies that matched our selection criteria. We characterized the studies within our review and performed a qualitative and quantitative analysis of the extracted meta-data regarding the climatic effects likely to change in these regions, including projected warming, early snowmelt, changes in precipitation, nutrient availability and their effects on seed maturation, seed dormancy, germination, seedling emergence and seedling establishment. Although the studies showed high variability in their methods and studied species, the qualitative and quantitative analysis of the extracted data allowed us to detect existing patterns and knowledge gaps. For example, warming temperatures seemed to favor all studied life stages except seedling establishment, a decrease in precipitation had a strong negative effect on seed stages and, surprisingly, early snowmelt had a neutral effect on seed dormancy and germination but a positive effect on seedling establishment. For some of the studied life stages, data within the literature were too limited to identify a precise effect. There is still a need for investigations that increase our understanding of the climate change impacts on high altitude and high latitude plants’ reproductive processes, as this is crucial for plant conservation and evidence-based management of these environments. Finally, we make recommendations for further research based on the identified knowledge gaps.

## 1. Introduction

Human-induced rising levels of greenhouse gases have already changed the global climate, altered natural systems’ function, and amplified extreme weather events [1,2]. The vulnerability of ecosystems to climate change is related to their sensitivity to environmental factors. High altitude (alpine, subalpine, treeline) and high latitude (tundra) ecosystems are considered among the most vulnerable to climate change because they are geographically restricted to areas characterized by low temperatures and in general, their species’ ecology is strongly influenced by the climatic parameters that are changing [3,4,5,6,7].

The consequences of climate change in high altitude and high latitude environments are already well documented; greater increases in temperatures compared with other parts of the world [7], changes in precipitation patterns [8,9], reductions in the depth and duration of snow cover during winter [7,8], and the decline of glaciers and permafrost [7,10,11], all of which are leading to changes in plant life cycles and the structure and diversity of plant communities [4,5,6,12].

Environmental changes and extreme climatic events during critical phases of plant life can profoundly affect species recruitment, growth, survival and distribution [13]. In this context, the early life-history stages of plants, such as seed germination and seedling establishment, are considered highly vulnerable to climate change as they are strongly influenced by climatic factors [14,15,16].

Temperature and water availability, two of the changing environmental factors, are the main drivers of the early life-history stages of high altitude and high latitude plants [17]. Before germinating, many alpine and arctic seeds need to experience cold temperatures (a cold stratification), generally provided during winter under the snow [17,18,19,20]. Soil warming above ambient temperatures, combined with soil moisture availability after the snowmelt, may trigger the germination of many species [20,21,22]. Rapid germination in early spring allows seedlings to establish in the most favorable part of the year [17,21]. However, amplified environmental changes and more recurrent extreme climatic events as a consequence of climate change will affect these plant life stages and could represent a bottleneck to future recruitment, thereby endangering the success of future plant populations [13,14].

Only recently have the effects of climate change on the early life-history stages of plants from high altitude and high latitude environments become a focus of researchers [23]. Historically it was believed that sexual reproduction in these ecosystems was rare and less important than clonal reproduction because of the harsh environmental conditions [24]. However, recent studies have found important persistent soil seed banks [25,26], high rates of natural seedling recruitment [27,28] and considerable gene flow among populations [29,30], which suggests that recruitment from seed is common and plays an essential role in high altitude and high latitude community dynamics [23].

To date, there has been no systematic review or meta-analysis of the effects of climate change on the early life-history stages of high altitude and high latitude plants. Existing reviews and meta-analysis in the topic, while valuable, have not been systematic in their approach [23], have not included analyses of the meta-data from previously published articles [31,32], nor have they included ecosystems above the treeline [33]. Yet, the knowledge gaps that are revealed in a systematic review of this nature, and the new insights generated from a meta-analysis, are critical for increasing our understanding of the regeneration potential, the threats to growth and survival, and the management implications of these environments under predicted future climate change scenarios.

Here, we investigated the literature and synthesized our current knowledge of how the early life-history stages of plants are impacted by changing climate parameters in high altitude and high latitude environments. A systematic search of articles published in academic journals on this topic and a qualitative and quantitate analysis of the meta-data were performed in order to answer five specific questions: (1) where were the studies conducted; (2) what species were studied; (3) what methods are used by researchers; (4) what climate change scenarios were simulated; (5) what is the overall mean effect of environmental changes on the early life-history stages of high altitude and high latitude plants?

## 2. Materials and Methods

### 2.1. Literature Search and Selection Criteria

We used the Systematic Quantitative Literature Review method [34] to search for studies regarding the effect of climate change in seeds and seedlings from alpine, treeline, subalpine and tundra ecosystems. We focused on studies that aimed to modify the various environmental factors that affect plants’ early life-history stages. In order to ensure transparent and the complete reporting of our systematic review and meta-analysis, we followed the Preferred Reporting Items in Systematic Reviews and Meta-Analyses (PRISMA) framework [35] (Checklist S1).

We searched for studies in four databases: Web of Science, Scopus, Science Direct, and Jstor (up to February 2020). We selected these databases because they are the most commonly used for ecology journals. We used the following string of keywords to find as many articles as possible that would help answer our research questions: (seed* OR germination OR establishment OR recruitment OR regeneration) AND (alpine OR subalpine OR tundra OR arctic OR Antarctic OR mountain OR treeline) AND (“climate change”) AND (temperature OR drought OR snow* OR warming OR fire). We adapted the keyword string syntax appropriately for each database. The search was limited to title, abstract and author-specified keywords. Furthermore, we used the reference lists of the articles obtained through the database searches to look for additional articles that matched our search criteria.

**Figure 1:**
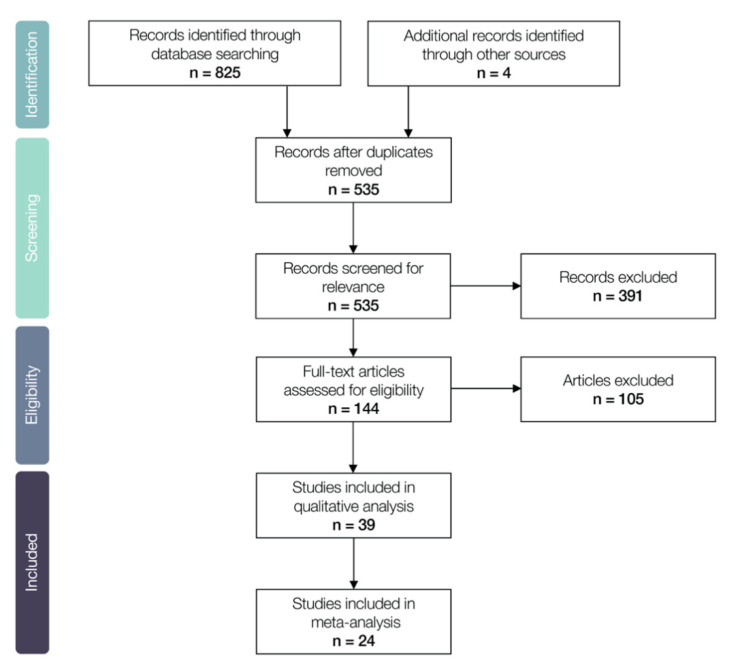
Preferred Reporting Items in Systematic Reviews and Meta-Analyses (PRISMA) flow diagram showing the number of articles identified, included and excluded through the different phases of our systematic review and meta-analysis.

We screened the obtained articles to ensure their relevance in a two-step selection process. In the first step, we read titles and abstracts and excluded articles that did not comply with our selection criteria. Articles needed to have: (a) a focus on the early stages of plant life (including seed maturation, seed dormancy, seed germination, seedling emergence and/or seedling establishment); (b) a focus on plant species naturally occurring at high altitude (alpine, subalpine and/or alpine treeline) and/or high latitude ecosystems (tundra and sub-tundra); and (c) articles that assessed the possible effects of climate change in these plant life stages. The second step involved obtaining the full text and reading the entirety of the articles and adding them to our database for review. At this step, articles were discarded if they: (a) did not include original research data; (b) had not subjected seeds or seedlings to appropriate controls and/or treatments that simulated a future climate or endeavored to alter environmental conditions; (c) had not been peer-reviewed, and (d) had not been entirely published in either English or Spanish. Thus, we did not include review and meta-analysis articles, government reports or grey literature. We kept all records of the literature search and the number of included and excluded articles in each step of this study according to the PRISMA framework (Figure 1).

### 2.2. Characterization of Studies

We recorded the relevant information for each article that fit the pre-determined selection criteria in a database. The database included the following categories: (a) details of publication: authors, title, year of publication, journal published in, plant life stage, study type (laboratory, in situ experiments or observational) and the modified environmental factor; (b) geographic location: continent, country, biome (alpine, subalpine, tundra, treeline) and habitat; (c) species: number of species, life form and distribution (native or exotic); (d) detail of the methods: the origin of the biological material used, seed post-harvest conditions, pre-germination treatments, experiment conditions and detail of the treatments (Table S1 and Database S1).

With the extracted data, and using Prism 8 and Adobe Illustrator, we constructed diagrams, tables, maps and charts to describe the literature search results, temporal and geographic occurrence of the articles, the ecosystems and species studied, the climate change scenarios simulated, and the methods used in the articles.

We also used VOSviewer software [36] to perform a citation analysis of the articles included in our systematic review and construct a citation map, where the relatedness of the articles was determined based on the number of times they cited each other. For this, we used CrossRef as an API source and the unit of the analysis was the articles. We used the association strength method and a minimum cluster size of ten to construct the citation map.

### 2.3. Effects of Climate Change on the Early Life-History Stages of High Altitude and High Latitude Plants

To determine the overall effect of climate change on the early life-history stages of high altitude and high latitude plants, we performed a qualitative and quantitative analysis of the reported results within the articles included in our review.

#### 2.3.1. Qualitative Analysis

To determine the effects of the treatments simulating a future climate within the studies included in our review, we looked for the direction and significance level from statistical analyses reported in each study and we recorded them in a database as a positive, negative or neutral effect. Then, we categorized the extracted observations according to the focused life stage and the climate change scenario to which they were subjected. With the extracted data, we constructed bar and pie charts.

In order to categorize and analyze the studies according to the life stage in which they focused, we defined the following life stages: (1) seed maturation, when fruits or seeds are still attached to adult plant; (2) seed dormancy, when fruits or seeds have been dispersed or collected from the adult plant but before ambient conditions meet the requirements for germination and during the time seeds are part of the soil seed bank; (3) seed germination, when ambient conditions meet the requirements for germination (radicle emerge from seed); (4) seedling emergence, when cotyledons open or emerge from the ground; (5) seedling establishment, after seedlings have emerged up until the end of the study and including all reported measurements of seedling survival, mortality or establishment.

While extracting the data for the qualitative analysis, we used the following criteria: (a) when the studies reported a single control and several treatments (e.g., temperature: control, +5 °C, +10 °C, +15 °C, and +20 °C), we recorded the results of the control and the treatment that best fits the climate change projections cited by the authors; and (b) when studies tested seeds from different provenances, we specifically recorded information for those seeds collected from either treeline, tundra, alpine or subalpine ecosystems (Database S1).

#### 2.3.2. Meta-Analysis

Within the articles of our review, we further selected those that: (a) included studies that have used distinct treatments and controls (we define “control” as the actual environmental conditions and “treatments” as the future environmental conditions under a particular climate change scenario); and (b) provided a statistical mean and a measure of the dispersion of the observations.

We categorized these observations according to the life stage and the climate change scenario to which they were subjected. For each observation, we extracted the mean, dispersion measurements, and number of replicates from controls and treatments. Data were extracted from the main text, tables, figures, or supplementary data of the articles. When the data were shown only visually in figures, we used the software ImageJ [37] to extract data points. We used the same criteria specified for the qualitative analysis to select the extracted data when the studies reported several treatments or multiple origins of seeds. We do not differentiate between lab and field studies since it has been determined that meta-analyses are unlikely to be biased by differences between ecological lab and field studies [38].

Then, we calculated the standardized mean difference as Hedges’ *g* for each observation and we ran a random-effects model to determine if the combined effect size for each life stage and climate change scenario combination was significantly different to zero (testing the hypothesis that the treatments simulating future climate had an effect). We omitted from our analysis the combinations with less than five observations as the correction for small samples of Hedges’ *g* could be diminished below this level [39]. Then, we constructed a forest plot to show the combined effect size for each analyzed combination.

#### 2.3.3. Study Heterogeneity and Publication Bias

To test for the level of variation among effect sizes included in the meta-analysis, we conducted a *Q* heterogeneity test (*I*^2^). To test for publication bias in our datasets (full dataset and in climate change scenarios datasets) we use two methods: (1) we constructed funnel plots and visually inspected them for asymmetry, and (2) we conducted the Egger’s regression test for funnel plot asymmetry.

## 3. Results

### 3.1. Literature Search

Our literature search recovered 825 articles. We found four more articles through other sources. After eliminating duplicates, we were left with 535 articles that were potentially relevant to answer our specific research questions. During the first phase of screening, and after reading the title and abstracts of the obtained articles, we excluded another 391 articles which failed to meet the established criteria. Throughout the second screening phase (reading whole articles) we excluded another 105 records for the same reasons. The number of studies included in the qualitative analysis was 39 and the included in the meta-analysis was 24 (Figure 1). The details of the included studies are provided in Reference List S1.

### 3.2. Characterization of Studies

#### 3.2.1. Publication Metrics

Articles included in our analysis were published during a 25-year period (1995–2020). The number of publications increased over time: two were published during 1995–2000, seven during 2000–2010 and 30 from 2010 to 2020 (Figure S1). Articles were published in 23 different journals, the most common journals publishing this kind of research being: *Global Change Biology* (*n* = 5, 12.1%), *Annals of Botany* (*n* = 4, 9.7%), *Plant Ecology* (*n* = 4, 9.7%) and *Oecologia* (*n* = 3, 7.3%). All other journals published two or less articles relative to our search. All articles were published in English, we did not find any articles in Spanish that met our criteria.

The citation analysis grouped 38 of the included articles (97%) in three clusters. Surprisingly, one article was not related to any other article included in our review (Figure S1).

#### 3.2.2. Geographic Distribution

**Figure 2:**
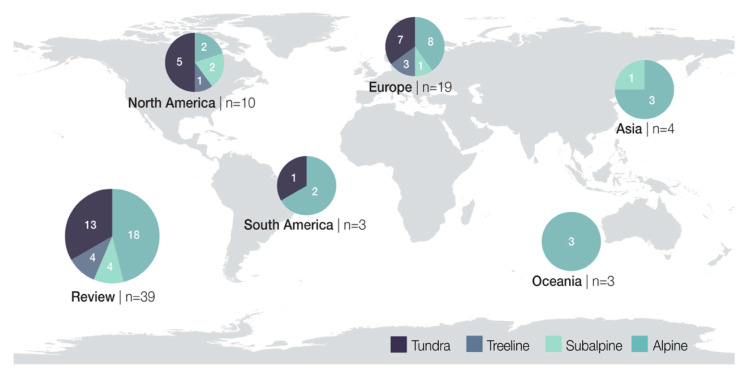
Geographic and ecosystem distribution of studies (within articles) that investigated the effects of climate change on early life-history stages of high altitude and high latitude plants.

The majority of the studies (within articles) were conducted in Europe (*n* = 19, 48.7%), with the most studies being conducted in Norway (*n* = 6) and Italy (*n* = 5), while studies from Sweden (*n* = 3), France (*n* = 2), Spain (*n* = 2) and Austria (*n* = 1) contributed to a lesser extent. Additionally, a large number of studies took place in North America (*n* = 10, 25.9%) with the most being from the United States of America (*n* = 6), Canada (*n* = 2) and Greenland (*n* = 2). The rest of the studies were conducted in Asia (*n* = 4, 10.2%: China *n* = 2, South Korea *n* = 1, Japan *n* = 1), South America (*n* = 3, 7.6%: Chile *n* = 2, Argentina *n* = 1) and Oceania (*n* = 3, 7.6%: Australia *n* = 3). No studies were recorded for Africa or Antarctica (Figure 2).

#### 3.2.3. Ecosystems, Plant Communities and Species

Most studies focused on species from alpine (*n* = 18, 47%) and tundra (*n* = 13, 33%) ecosystems. The remaining studies focused on species from treeline (*n* = 4, 10%) and subalpine ecosystems (*n* = 4, 10%, Figure 2).

Regarding the plant community where the studied species came from, tundra forest and snowbeds were well represented, with nine and five studies, respectively. The plant communities that contributed to a lesser extent were subalpine forest (*n* = 4), treeline forest (*n* = 4), alpine meadow (*n* = 4), heathlands (*n* = 3), grasslands (*n* = 2), glacier foreland (*n* = 2), and herbfield (*n* = 2). Four studies did not specify the particular plant community where they were carried out.

Overall, seeds and seedlings from 246 species across 143 genera and 37 families were assessed to determine their responses to future climate conditions. There were 171 forbs, 33 graminoids, 23 shrubs and 19 trees. According to the authors, all species were native to the region where they were collected and studied. The taxonomic identity of the species included in our review is provided in Table S2.

#### 3.2.4. Climate Change Scenarios and Early Life-History Stages

**Figure 3:**
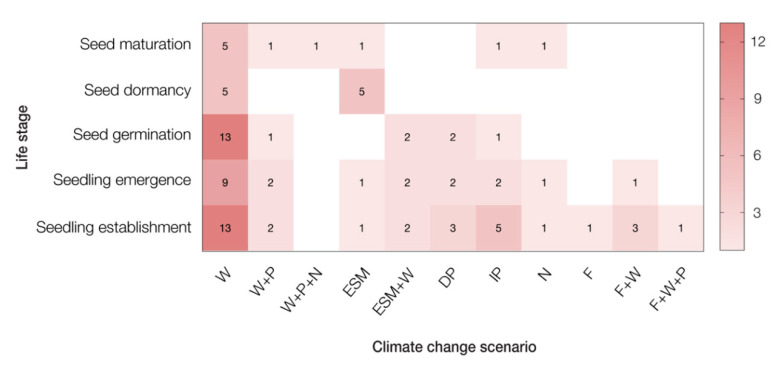
Counts of the number of instances an early life-history stage of plants was studied under a particular climate change scenario. The sum of frequencies could be more than the number of studies included in our review (*n* = 39), because more than one life stage or climate change scenario may have been tested in each study. W: warming, W+IP: warming and increase in precipitation, W+IP+N: warming, increase in precipitation and nutrient availability, ESM: early snowmelt, ESM+W: early snowmelt + warming, DP: decrease in precipitation, IP: increase in precipitation, N: nutrient availability, F: post-fire conditions, F+W: post-fire and warming, F+W+P: post-fire, warming and precipitation.

Of the total studies included in our review, 14 focused on seeds, 10 on seedlings and 15 on both life-history stages. The environmental factors that were most frequently modified within the studies were temperature (*n* = 35), water availability (*n* = 14), snow cover (*n* = 10) and nutrient availability (*n* = 5). Combining these frequently modified environmental factors and post-fire conditions (*n* = 3), as a major environmental perturbation, the studies simulated a total of 11 different future climate change scenarios (Figure 3). Not all the scenarios were included in the qualitative and quantitative analysis because not all studies provided sufficient data or had established appropriate controls.

According to the most accepted climate change projections in high altitude and high latitude ecosystems, the majority of the studies within our review manipulated environmental factors in a realistic scenario (Table 1). However, only 40% of the studies mentioned whether the climate change projection for their region was taken into account during their experimental design.

#### 3.2.5. Methods and Experimental Conditions

Of the studies included in our review, 53% (*n* = 21) were field experiments, 30% (*n* = 11) laboratory experiments and 17% (*n* = 7) used a combination of both methods (Table 1). Of the total field studies, 52% (*n* = 11) specified the soil type within the experiment site and 76% (*n* = 16) specified the particular substrate where seeds and seedlings were established (Database S1).

### 3.3. Effects of Climate Change on the Early Life-History Stages of High Altitude and High Latitude Plants

**Figure 4:**
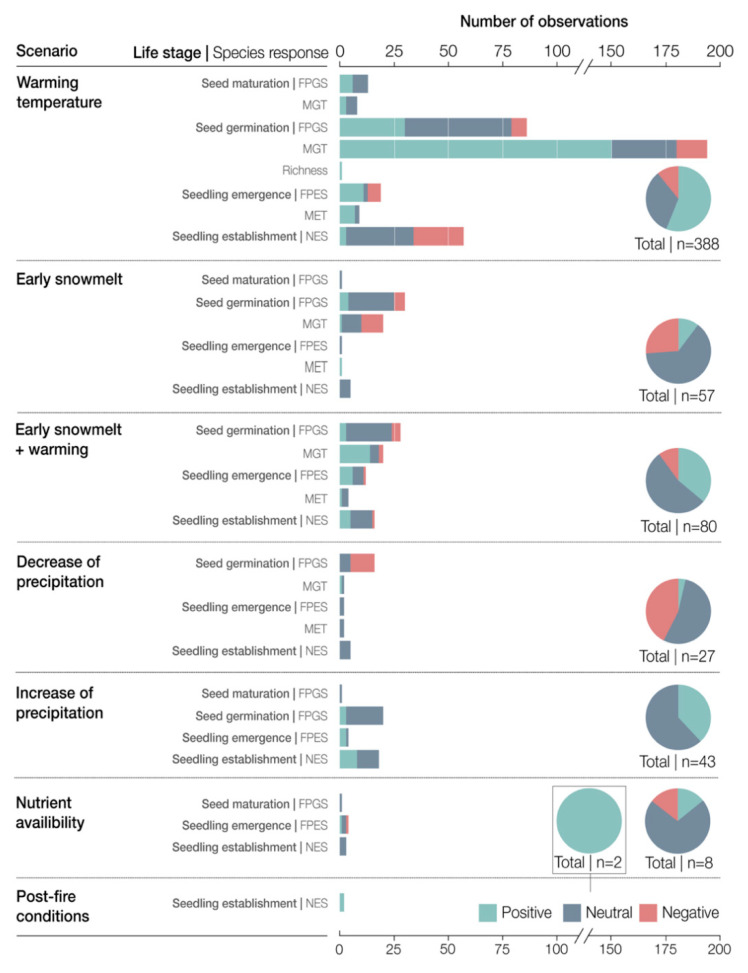
Distribution of positive, negative and neutral observations reported by the studies included in our review regarding the effects of climate change on early life-history stages of high altitude and high latitude plants. FPGS: final proportion of germinated seeds, MGT: mean germination time, FPES, final proportion of emerged seedlings, MET: mean emergence time, NES: number of established seedlings.

The dataset used for the qualitative analysis included 605 observations: 263 positive, 257 neutral and 85 negatives. From the total of the observations, 24 were for seed maturation, 50 for seed dormancy, 366 for seed germination, 79 for seedling emergence and 86 for seedling establishment (Database S1). We categorized the observations with respect to the following climate change scenarios: warming temperatures, changes in the snow cover, changes in precipitation, nutrient enhancement and post-fire conditions. This resulted in 22 combinations of climate change scenarios and plant life-stages and 32 combinations of climate change scenarios and particular plant responses (Figure 4).

The dataset used for the meta-analysis included 290 observations: 161 for temperature, 77 for snow cover, 28 for increase in precipitation and 24 for decrease in precipitation (Database S1). Many studies from the qualitative analysis were excluded from the meta-analysis (*n* = 15) because they did not provide data to calculate effect sizes.

#### 3.3.1. Warming Temperature

**Figure 5:**
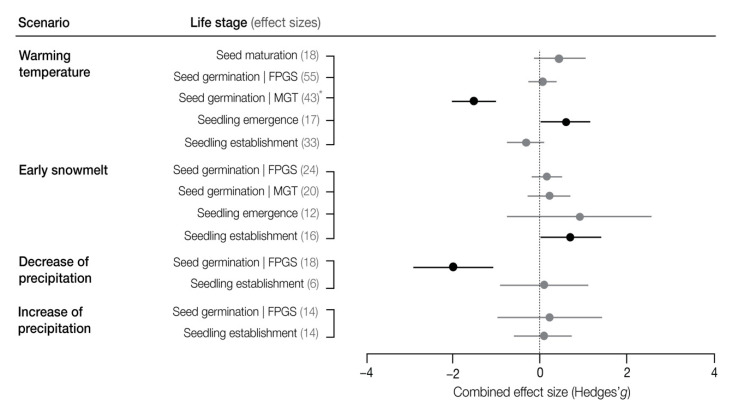
Combined effect sizes (Hedges’g and ±95% confidence intervals) of the impacts of climate change scenarios on early life-history stages of high altitude and high latitude plants. Black dots indicate a statistically significant meta-analytic effect. Gray dots indicate that the combined effect is not significantly different from zero. * Observe that the combined effect size for mean germination time on the negative side of zero, was interpreted as a positive effect in the results and discussion. FPGS: final proportion of germinated seeds, MGT: mean germination time.

The increase in temperature was the most simulated climate change scenario within the articles of our review (*n* = 35). Overall, warming temperatures had mostly a positive effect on the early life-history stages of high altitude and high latitude plants (54% of the observations in the qualitative analysis, Figure 4). Warming temperatures during seed maturation seemed to have mostly neutral effects on the final proportion of germinated seeds (FPGS, 7 out of 13 observations in qualitative analysis, Figure 4) and on the mean germination time (MGT, five of eight observations in qualitative analysis, Figure 4). This lack of effect was confirmed by the meta-analysis (*p* = 0.067, *n* = 18 observations from seven studies, Figure 5). Warmer temperatures during germination mostly reduced (positive effect) the mean germination time (149 of the 193 observations in qualitative analysis; Figure 4) but had a mostly neutral effect on the FPGS (49 of the 86 observations in qualitative analysis, Figure 4). This was confirmed by the meta-analysis (*p* = 0.001, *n* = 38 observations from six studies for MGT and *p* = 0.111, *n* = 53 observations from eight studies for FPGS, Figure 5).

The mean seedling emergence time and the final proportion of emerged seedlings was positively affected by temperature in the qualitative analysis (11 of 21 and 7 of 9 observations in qualitative analysis, respectively, Figure 4); however, this effect was non-significant when analyzed in the meta-analysis (*p* = 0.285, *n* = 17 observations from six studies, Figure 5). Warmer temperatures seemed to have a mostly neutral effect on seedling establishment (31 of 57 observations in the qualitative analysis, Figure 4), which was confirmed by the lack of significant results in the meta-analysis (*p* = 0.134, *n* = 30 observations from 10 studies, Figure 5).

#### 3.3.2. Changes in Snow Cover

The studies within our analysis that manipulated the duration of snow cover, reported mostly neutral treatment effects (63% of the observations in the qualitative analysis, Figure 4). Early snowmelt during germination appeared to mostly increase the mean germination time (10 of the 19 observations in qualitative analysis, Figure 4) but have a neutral effect on the FPGS (21 of the 30 observations in qualitative analysis, Figure 4). Early snowmelt also appeared to have neutral effects on seedling establishment (five out of five observations in the qualitative analysis, Figure 4). Only one observation was reported on the effects of early snowmelt on seed maturation and hence no meta-analysis of this data was performed.

When an early snowmelt treatment was followed by an increase in temperature, the effect on the FPGS remained neutral (21 of 28 observations) but caused an overall change in MGT from negative to positive (14 of 20 observations in qualitative analysis), although the meta-analysis revealed a non-significant effect (*p* = 0.105, *n* = 45 observations from three studies, Figure 5). Seedling emergence appeared to respond positively to a combination of early snowmelt and warming (nine of 16 observations in qualitative analysis, Figure 4), but again, this response was not significant (*p* = 0.38, *n* = 16 observations from three studies, Figure 5). Finally, seedling establishment responded significantly positively when early snowmelt was followed by warmer temperatures (8 out of 13 observations in the qualitative analysis and *p* = 0.019, *n* = 16 observations from three studies in the meta-analysis, Figure 4 and Figure 5).

#### 3.3.3. Changes in Precipitation

The studies that simulated a decrease in precipitation during the early life-history stages of high-altitude and high latitude plants, reported mostly neutral effects of their treatments (55% of the observations in the qualitative analysis, Figure 4). However, the FPGS respond negatively to a decrease in precipitation (11 of 16 observations in qualitative analysis, Figure 4), which was confirmed by the meta-analysis (*p* = 0.001, *n* = 16 from two studies, Figure 5). The effect of a decrease in precipitation appeared to have neutral effects on seedling establishment (five of five observations in qualitative analysis, Figure 4), and no effect was found in the meta-analysis (*p* = 0.394, *n* = 6 from two studies, Figure 5). Very few studies reported observations for MGT (*n* = 1) and seedling emergence (*n* = 1), and hence no attempt was made to infer an overall effect or perform a meta-analysis for these data. No studies were found that investigated the effects of a decrease in precipitation on seed maturation and seed dormancy.

Studies that focused on the effects of increases in precipitation on the early life-history stages of high altitude and high latitude plants reported mostly neutral and positive effects. For seed germination, an increase in precipitation showed predominantly neutral effects in the qualitative analysis (17 of 20 observations, Figure 4) and a lack of significance in the meta-analysis (*p* = 0.356, *n* = 14 observations from 2 studies, Figure 5). Seedling emergence appeared to respond positively to an increase in precipitation (three of four observations in the qualitative analysis). Seedling establishment showed a neutral effect in the qualitative analysis (eight of 15 observations) and meta-analysis (*p* = 0.395, *n* = 14 observations form two studies, Figure 5). Very few observations were reported for seed maturation (*n* = 1) and hence an overall effect and meta-analysis was not performed. No studies were found that investigated the effects of an increase in precipitation on seed dormancy.

#### 3.3.4. Nutrient Availability

Higher nutrient availability seemed to have mostly neutral effects on seed maturation, seedling emergence and seedling establishment (one of one, two of four and two of two observations, respectively, Figure 4); however, there were too few observations and studies available to perform a meta-analysis. No studies were found that investigated the effects of nutrient availability on seed dormancy and seed germination.

#### 3.3.5. Post-Fire Conditions

Very few studies (*n* = 3) investigated the effects of post-fire conditions in the early life-history stages of high altitude and high latitude plants. Additionally, from these, only one study established an appropriate control for the post-fire conditions treatment (unburnt areas). Very few observations were recorded (*n* = 2) to infer an overall effect or to perform a formal meta-analysis. No studies were found that investigated the effects of fire on seed maturation, seed dormancy, seed germination or seedling emergence.

#### 3.3.6. Heterogeneity and Publication Bias

The heterogeneity test indicated a value of 76.06% for *I*^2^ for the full meta-analysis dataset, 76.14% for warming, 66.59% snow cover, 78.31% for water decrease and 71.65% for water increase datasets (all *p* < 0.001). The values of *I*^2^ suggest a medium and high level of estimated heterogeneity within all datasets.

We detected evidence of publication bias in the full dataset and the environmental variable datasets towards negative values. The exceptions to this trend were the snow cover dataset, which showed a bias towards positive values, and the precipitation dataset, which was symmetrical (Figure S2). The Egger’s regression test confirmed the funnel plot trends (*z* = −6.7, *p* < 0.001 for the full dataset; *z* = −9.57, *p* < 0.001 for the temperature dataset; *z* = 5.2 *p* < 0.001 for the snow cover dataset, *z* = −6.2, *p* < 0.001 for the decrease in precipitation dataset; and *z* = 1.49, *p* = 0.134 for the increase in precipitation dataset).

## 4. Discussion

We have integrated the responses to climate change of high altitude and high latitude plants during their early life-history stages using a systematic review approach and a subsequent analysis of the meta-data from previously published studies. The results showed considerable heterogeneity in plant responses to changing environmental variables as a consequence of the wide variation of species and plant life forms studied within the articles of our review; however, the qualitative and quantitative analyses have allowed us to identify general patterns for some life stages and climate change combinations. For other plant life stages, data within the literature are too limited to detect a precise effect.

The early life-history stages are critical in plant life cycles and are considered highly vulnerable to climate change [14]. As such, their responses to a predicted future climate at high altitude and high latitude environments have been reported and discussed before in the literature [14,17,23,40]. The citation analysis pointed to Shevtsova et al. [41], Milbau et al. [22], Graae et al. [42] and Mondini et al. [21] as the more influential articles within our network (including only citations made by papers from our review). The research of Wookey et al. [43] and Milbau et al. [22] were the most influential when we took into account the total citations received. The majority of the studies included in our review were published during the last decade, indicating an increase in interest by scientists for a topic that was once reported as being largely neglected [13].

However, our results suggest that there is still a lack of primary studies investigating the interactions between some early life-history stages of plants and some environmental factors that are predicted to change in the future. More observations regarding seedling emergence and establishment have been made than seed maturation, seed dormancy and seed germination. Regarding the environmental factors likely to change, an increase in temperature is the most studied scenario within the articles that were part of our review. This is likely because the predicted changes in temperature at high altitude and high latitude ecosystems are expected to be greater there than in other biomes [7]. In contrast, the effects of water availability, nutrient enhancement or post-fire conditions are poorly studied.

Our results also suggest a strong geographic bias; the majority of the studies took place in the northern hemisphere, specifically in Europe and North America. Consequently, there is limited knowledge regarding the threats of climate change on the early life-history stages of alpine, tundra and treeline plants from other regions, such as Asia, Central and South America and Africa. There is also a lack of studies regarding the early life-history stages of tropical alpine species. These findings partially contrast with the reported trend for global alpine research, where most of the studies during the last decade occurred in Asia, particularly in China, but coincide with the lack of studies from the southern hemisphere [44] or tropical alpine regions [20].

Regarding the methods used by the studies included in our review, we found a great variation in the location (field or controlled conditions), instruments used, soil and substrate types, time scale and intensity in which the seeds, seedlings and environmental factors were manipulated (Table 1). The wide diversity in the methods used in some of the studies included in our review have been discussed before [32]. Furthermore, the effectiveness of some of the methods used to modify environmental variables within the studies of our review and the complexity in climate change manipulation experiments have also been discussed before, for example, the unwanted side effects of open-top chambers on light, temperature and wind [45,46,47].

There were also inconsistencies in how the effect of different treatments on modified environmental variables were reported in various studies. For example, in studies where temperatures were modified, some reported air temperature or surface temperature, whereas only a few reported the upper soil layer temperature, a measure that would be of greater relevance to experiments where seeds are buried. In addition, in many studies where precipitation was modified, the authors generally only reported the amount of water added or restricted compared to control plots, whereas reporting soil moisture values or measures of plant-available water would have been more relevant to the responses of plants under those treatments, and would therefore have taken soil and substrate type, drainage and evaporation into consideration.

Despite the inherent variation between studies included in our review, all used an appropriate study design and successfully modified at least one environmental factor according to the accepted climatic scenarios for their particular region. This contrasts with some reviews that have reported studies which simulated unrealistic climate change scenarios [48].

### 4.1. Effects of Climate Change on the Early Life-History Stages of High Altitude and High Latitude Plants

#### 4.1.1. Warming Temperature

Temperature is a critical driver for the early life-history stages of plants [14] and is expected to increase by a greater magnitude at high altitude and at high latitude ecosystems than in other parts of the world [7]. The reviewed studies collectively agreed that the expected warming temperatures would act to promote all early life-history stages, except for seedling establishment (Figure 4).

The positive and neutral effects we found in the FPGS and MGT in response to warming coincide with the reported positive role of warmer temperatures in the arctic and alpine plants reproductive success [31] and as a key driver for alpine plants’ germination [20]. Interestingly, in the only soil seed bank experiment within our review, soil warming reduced the total number of germinants; however, this reduction was caused by the response of a single abundant species, which suggests a species-specific response to this climate change scenario [26]. Our findings suggest that warming temperatures will benefit from germination and reduce mean germination time in high altitude and high latitude environments; however, factors such as species-specific responses, and other local environmental conditions, such as soil moisture, also need to be considered.

Positive and neutral effects of warming temperatures on the number of emerged seedlings were reported for tundra, treeline, alpine snowbed and sub-alpine species [49,50,51,52]; however, a negative effect was reported for some arctic and alpine forbs in studies where authors suggested species-specific responses [53,54]. The mean seedling emergence time was mostly reduced on treeline, subalpine and alpine species [51,55,56], but a neutral effect was reported for a mat-forming perennial plant on the arctic tundra [54].

We report the overall reduction in mean germination time and seedling emergence time as a positive effect of warmer temperatures, as it will increase the time available during the growing season for seedling establishment. However, this could be interpreted as a negative effect if warming temperatures are preceded by early snowmelt and seedlings are exposed to early spring frost events or a longer growing season could potentially put emerging seedlings into a drought-stress situation if the time between snowmelt and summer-autumn rains is prolonged [17,41,49]. Regardless of these possible scenarios previously discussed, the reported effects of warming temperature on seedling establishment were inconsistent, which has also been reported for the treeline ecosystem [33]. The variation in seedlings’ responses among the studies of our review, suggests that the effects of temperature may be species-specific or depend on local environmental conditions, such as the degree of warming or the water availability during warmer periods.

#### 4.1.2. Changes in Snow Cover

Changes in snow cover depth and duration are some of the most evident effects of climate change at high altitude and high latitude ecosystems [7]; however, studies that manipulated the duration of the snow cover within our review reported mostly neutral effects. During the germination period, early snowmelt appeared to mostly increase the mean germination time of European tundra species, but this effect was diminished when warming temperatures followed snowmelt [22]. Unexpectantly, treatments simulating a reduction in snow cover had mostly neutral effects on the final proportion of germinated seeds among studies of our qualitative analysis, which contrasts with the strong need for cold stratification of seeds under snow during winter before a spring germination, as reported for several tundra and alpine species [14,17,20]. In contrast, seedling emergence and seedling establishment were mostly positively affected by early snowmelt followed by warmer temperatures and the meta-analysis confirmed this for seedling establishment rates (Figure 5). This positive effect detected by the meta-analysis may have been strongly influenced by the response of glacier foreland forb species, which benefit from the extension of the snow-free period in spring, thereby increasing seedling establishment and providing greater resistance to summer drought and to next winter extremes [57].

#### 4.1.3. Changes in Precipitation

Soil moisture availability (mainly via precipitation), combined with temperature, are critical drivers for the success of the early life-history stages in cold-climate plants [14]. The frequency and intensity of precipitation differs worldwide, and as a consequence, projections for future annual precipitation also vary according to each region, for example, an increase is predicted for the Himalaya region, East Asia and the European Alps and a decrease for the Southern Andes and the Australian Alps [8].

Experimental drier conditions during laboratory [56] and field [58] studies, found a negative effect on the final proportion of germinated seeds from subalpine species. This negative effect was subsequently confirmed by our meta-analysis (Figure 5). The final proportion of germinated seeds can be used as an indicator of recruitment success, and the reported sensitivity of species to drought may indicate major implications for their persistence. However, when the effects of a decrease in soil moisture availability were tested on the seedlings of five subalpine tree species, only neutral effects were reported [52,56,59]. Our findings suggest that seedling stages (such as establishment and survival) are not as affected as seed germination. Further studies are needed to investigate the effects of a drier future during seed stages, such as seed maturation, seed dormancy and soil seed banks as they could represent a bottleneck for future plant recruitment.

When an increase in precipitation was crossed with warmer temperatures to determine seed germination rates in the European tundra, mostly neutral effects were reported [41]. In two studies, where natural gradients were utilized to simulate a wetter future [53,60], seedling emergence appeared to respond positively to an increase in precipitation; however, the meta-analysis showed no significant effects. For studies in our review that measured seedling establishment under wetter conditions [41,53,60,61,62,63], neutral and positive effects were reported (Figure 4); however, the meta-analysis showed no significant effects. This variation detected in the meta-data is probably a response to the different life forms of the species included in the datasets. We found that almost all the included tree species showed a positive effect to a wetter environment (except for one observation), whereas other life forms (forbs, graminoids and shrubs) showed mostly neutral effects (Database S1). Thus, the responses to increases in precipitation among high altitude and high latitude species cannot be generalized and further investigations into the variation in responses of different life forms are warranted.

#### 4.1.4. Nutrient Availability

Nutrient availability and uptake in high altitude and high latitude ecosystems is limited by low temperatures [64]. Therefore, as a consequence of future warming, nutrient availability in these ecosystems is expected to increase due to faster mineralization of the soil organic matter [65] and an increase in atmospheric nitrogen deposition [66]. This nutrient enhancement may lead to an increased assimilation of nutrients by plants [64] and thus, may have flow-on effects to the success of seed germination and seedling survival. Overall, the studies within our review reported neutral effects of nutrient additions on the early life-history stages; however, many different species-specific responses had been reported. For example, nutrient enhancement had contrasting effects (positive, negative and neutral) on the seedling emergence and seedling establishment of three tundra species within the same study [49]. Similar contrasting effects were reported for alpine snow bed plants [67]. Over a three-year period, an increase in nutrient availability had a positive effect on the number of seeds produced but a neutral effect on the seed viability of *Dryas octopetala* in the Arctic tundra [43]. The lack of a clear effect on the response to nutrient additions may result from the low number of observations recorded in our review for this scenario (*n* = 6); too few to perform a formal meta-analysis of the data. Although the potential role of nitrogen and nitrate as triggers for germination have been reported [68], we have not found studies that directly recorded the effects of nutrient availability during germination (reported measures were always in relation to seedling emergence).

### 4.2. Heterogeneity and Publication Bias

Although we categorized the extracted observations according to the life stage and the climate change scenario to which they were subjected in order to reduce the responses variation and combine similar ecological responses in subgroup meta-analysis, we detected medium (50–75%) and high (>75%) levels of heterogeneity (*I*^2^) in our datasets. This variation could be explained by the differences in the methods and study designs of the articles included in our meta-analysis [38,69]. For example, “how” (e.g., open-top chambers, FATI technique or controlled glasshouses) and “how much” (e.g., +1, +2, or +5 °C vs. control) the studies modified the temperature or other environmental variables (see Table 1 for methods details). The heterogeneity could also be explained by the different ecosystems and species that were part of our database, each having its own autecology and responding differently to environmental stimuli.

The funnel plots and Egger’s test results showed evidence of publication bias in our datasets favoring positive treatments effects. This is probably due to such positive results being more likely to be published [70]. Furthermore, the publication bias could be explained by our decision to only look for articles published in English and Spanish, leading us to possibly exclude studies with inconclusive or non-significant results, as these studies are more frequently published in local journals using the local language [70]. Additionally, the bias may be a result of including only 60% of the studies from the systematic review in the meta-analysis. The remaining 40% of studies that we excluded (as they failed to report the necessary results according to our specifications), may have provided symmetry to the funnel plots. Therefore, the results from this review and any general conclusions drawn from them should be interpreted with caution.

### 4.3. Implications for Future Research

We found large knowledge gaps and inconsistencies among the studies that were part of our review. Based on these, we make the following recommendations for future research on the effects of climate change on the early life-history stages of plants at high altitude and high latitude ecosystems:

Firstly, the knowledge regarding the early life-history stages in which this review is focused, is unbalanced. There is a good understanding of how a future climate will affect seed germination, seedling emergence and seedling establishment. However, of the 603 observations in our qualitative analysis, only 19 observations focused on how future climate may affect seed maturation and only 30 observations focused on seed dormancy and the soil seed bank stages. Therefore, we suggest future research should address the possible effects of climate change on the responses of seeds to the climate parameters that are changing (white and pale red squares in Figure 3).

Secondly, the authors have reported on the effects of different climate parameters to various extents. To date, we can predict relatively well how warming temperatures will affect the early life-history stages of plants from alpine, tundra and treeline ecosystems. However, the effects of other environmental factors, that are also likely to change, such as nutrient availability [65,71] and the frequency and intensity of disturbances, such as fire [72,73], are poorly studied (white and pale red squares in Figure 3). There is also a lack of studies that test the effects of two or more environmental factors simultaneously, and how they amplify or diminish their own effects. Thus, more research is needed to improve our understanding of how these distinct future climate scenarios will affect the growth and survival of high altitude and latitude plants, which ultimately has flow-on ramifications for ecosystem processes and function.

Thirdly, we suggest that future field studies that aim to test the responses of seeds and seedlings under modified environmental conditions, report data for ground and underground temperature, rather than air temperature, and soil moisture values or plant available water, rather than the amount of water added or restricted. Additionally, because seeds and seedlings are in direct contact with the soil and substrate layer, we strongly recommend a detailed description of these factors when describing the experimental conditions. These additional data can then be used to better explain the environmental conditions under which buried seeds and small seedlings were tested.

Finally, there is a strong geographical bias in the studies included in our review. Only 15% of the studies of our review came from the southern hemisphere and none from tropical alpine regions, as also detected by other researchers [20,44]. Moreover, while the climate at treeline, above treeline in the alpine and in tundra ecosystems show many commonalities, there are also a number of different environmental features reflecting regional, rather than global patterns [17]. Thus, there is an urgent need for research in these underrepresented regions, such as in Central America, northern South America, Africa, and to a lesser extent Oceania and Asia, as it would be incorrect to assume that the reported effects in this review apply equally in all of these regions.

## 5. Conclusions

This systematic review and the meta-analysis synthesize current knowledge regarding the predicted climate change effects on the early life-history stages of plants in high altitude and high latitude environments. Our results showed an increase in the number of studies regarding this topic during the last decade. The data we extracted from the literature presented medium and high levels of heterogeneity, probably due to the variation in research methods, different studied plant life forms, local environmental factors, and studied species auto-ecology. However, the qualitative analysis and meta-analysis allowed us to detect existing general patterns and knowledge gaps. Warming temperatures seemed to favor all studied life stages except for seedling establishment. Four our surprise, changes in the snow cover showed mostly neutral effects, except for seedling establishment which was positively affected. A decrease in precipitation showed a strong negative effect on seed stages, while seedlings responded mostly neutrally to this scenario. An increase in precipitation had mostly neutral and positive effects; however, plant life forms responded differently. For some of the studied life stages, there is still a lack of understanding on how they will be affected by future climate (e.g., seed maturation and seed dormancy). There is also a lack of studies testing some of the predicted future climate change scenarios on seeds and seedlings (e.g., changes in precipitation, nutrient availability and post-fire conditions). There is still a crucial need for investigations that will increase our understanding of how the early life-history stages of high altitude and high latitude plants will cope with climate change, as this is crucial for plant conservation and the evidence-based management of these environments.

## Figures and Tables

**Table 1:** The particulars of methods used by the studies within our review. The count of the number of instances could be more than the number specified for each environmental variable because more than one method or environmental variable may have been tested in each study.

Environmental Variable	Particularities of the Methods	Categories	No. of Instances
Temperature	Where was modified?	Laboratory/Glasshouse	10
		Field	18
		Combination of both	7
	How was modified?	Growth chambers (laboratory)	11
		Temperature controlled glasshouse	2
		Open-top chambers and similar	11
		Infrared heaters and similar	5
		In situ glasshouse	3
		Natural gradient	3
	How much was modified vs. control?	+1 °C	7
		+2 °C	13
		+3 °C	2
		+4 °C	3
		+5 °C	6
		More than +5 °C	4
Snow cover	Where was modified?	Laboratory	4
		Field	4
		Combination of both	2
	How was modified?	Modifying temperature in growth chambers or similar (laboratory)	6
		Adding or removing snow	6
	How much was modified vs. control?	−4 months (all winter)	1
		−10 weeks	1
		−8 weeks	3
		−4 weeks	1
		−2 weeks	1
		+1 weeks	2
		+2 weeks	1
Water availability	Where was modified?	Laboratory	2
		Field	9
		Combination of both	3
	How was modified?	Water potential solutions	2
		Restricting rain or adding water	9
		Using a natural gradient	3
	How much was modified vs. control?	+50% mean annual precipitation	4
		+35% mean annual precipitation	1
		+20% mean annual precipitation	2
		+10% mean annual precipitation	2
		−20% mean annual precipitation	1
		−50% mean annual precipitation	2
		1 to 0.5 PEG solution concentration	2
		0.5 to 0.1 PEG solution concentration	2
Nutrient availability	Where?	Field	5
	How?	Adding nutrients	5
	How much?	1 to 5 g/per m^2^ year	2
		5 to 15 g/per m^2^ year	2
		>15 g/per m^2^ year	1
Post-fire conditions	Where?	Field	3
	How?	Natural post-fire conditions	3

## Data Availability

Data used in this study are available in the Supplementary Materials (Database S1) or by contacting J.V.-R. at jvazquezramirez@deakin.edu.au.

## Supplementary Materials

The following are available online at https://www.mdpi.com/article/10.3390/plants10040768/s1, Database S1: Systematic review and meta-analysis database. Reference List S1: Details of the publication included in our systematic review and meta-analysis, Table S1: Systematic review and meta-analysis proforma, Table S2: Taxonomic identity of species, Figure S1: Citation map, Figure S2: Funnel plots of datasets, and Checklist S1: PRISMA checklist.
