# Supplementary material for: Seeds and Seedlings in a Changing World: A Systematic Review and Meta-Analysis from High Altitude and High Latitude Ecosystems
Source: Plants (Basel). 2021 Apr 14;10(4):768. doi: 10.3390/plants10040768 (PMC8070808; doi:10.3390/plants10040768)
Supplement: Supplementary file 1 [file plants-10-00768-s001.zip › Supplementary information/Supplemetary information.docx]

Supplementary Information.

Article title: Seeds and seedling in a changing world: a systematic review and a meta-analysis from high altitude and high latitude ecosystems.
Authors: Jerónimo Vázquez-Ramírez and Susanna E. Venn

The following supplementary information is available for this article:

- **Reference List S1.** Publications included in our systematic review and meta-analysis.
- **Table S1.** Variables extracted from each article that is part of our systemic review, qualitative analysis and meta-analysis
- **Table S2.** Taxonomic identity (family, genus, species) and life form of the taxa studied within the articles that were part of our systematic review.
- **Figure S1.** Citation map of the articles included in our systematic review.
- **Figure S2.** Funnel plots for the datasets used in the meta-analysis.
- **Checklist S1.** PRISMA (Preferred Reporting Items in Systematic Reviews and Meta-Analyses) checklist
- **Database S1.** It can be found in a separate excel file. If you cannot find it, you can request it to first author at jvazquezramirez@deakin.edu.au or jero.vazquez@live.com

**Reference List S1.** Publications included in our systematic review and meta-analysis. The red asterisk (*) indicates studies included in the meta-analysis.

1. Bader, M. Y., Loranger, H., Zotz, G., & Mendieta-Leiva, G. (2017). Responses of tree seedlings near the alpine treeline to delayed snowmelt and reduced sky exposure. Forests, 9(1). https://doi.org/10.3390/f9010012 *
2. Bernareggi, G., Carbognani, M., Mondoni, A., & Petraglia, A. (2016). Seed dormancy and germination changes of snowbed species under climate warming: The role of pre- and post-dispersal temperatures. Annals of Botany, 118(3). https://doi.org/10.1093/aob/mcw125 *
3. Bernareggi, G., Carbognani, M., Petraglia, A., & Mondoni, A. (2015). Climate warming could increase seed longevity of alpine snowbed plants. Alpine Botany, 125(2), 69–78. https://doi.org/10.1007/s00035-015-0156-0 *
4. Camac, J. S., Williams, R. J., Wahren, C. H., Hoffmann, A. A., & Vesk, P. A. (2017). Climatic warming strengthens a positive feedback between alpine shrubs and fire. Global Change Biology, 23(8), 3249–3258. https://doi.org/10.1111/gcb.13614
5. Cooper, E. J., Dullinger, S., & Semenchuk, P. (2011). Late snowmelt delays plant development and results in lower reproductive success in the High Arctic. Plant Science, 180(1), 157–167. https://doi.org/10.1016/j.plantsci.2010.09.005 *
6. García-Fernández, A., Escudero, A., Lara-Romero, C., & Iriondo, J. M. (2015). Effects of the duration of cold stratification on early life stages of the Mediterranean alpine plant Silene ciliata. Plant Biology, 17(2), 344–350. https://doi.org/10.1111/plb.12226
7. Graae, B. J., Alsos, I. G., & Ejrnaes, R. (2008). The impact of temperature regimes on development, dormancy breaking and germination of dwarf shrub seeds from arctic, alpine and boreal sites. Plant Ecology, 198(2), 275–284. https://doi.org/10.1007/s11258-008-9403-4
8. Graae, B. J., Ejrnæs, R., Marchand, F. L., Milbau, A., Shevtsova, A., Beyens, L., & Nijs, I. (2009). The effect of an early-season short-term heat pulse on plant recruitment in the Arctic. Polar Biology, 32(8), 1117–1126. https://doi.org/10.1007/s00300-009-0608-3
9. Grau, O., Ninot, J. M., Cornelissen, J. H. C., & Callaghan, T. V. (2013). Similar tree seedling responses to shrubs and to simulated environmental changes at Pyrenean and subarctic treelines. Plant Ecology and Diversity, 6(3–4), 329–342. https://doi.org/10.1080/17550874.2013.810311 *
10. Han, A. R., Kim, H. J., Jung, J. Bin, & Park, P. S. (2018). Seed germination and initial seedling survival of the subalpine tree species, Picea jezoensis, on different forest floor substrates under elevated temperature. Forest Ecology and Management, 429(July), 579–588. https://doi.org/10.1016/j.foreco.2018.07.042 *
11. Hansen, W. D., & Turner, M. G. (2019). Origins of abrupt change? Postfire subalpine conifer regeneration declines nonlinearly with warming and drying. Ecological Monographs, 89(1). https://doi.org/10.1002/ecm.1340 *
12. Hobbie, S. E., & Chapin, F. S. (1998). An experimental test of limits to tree establishment in Arctic tundra. Journal of Ecology, 86(3), 449–461. https://doi.org/10.1046/j.1365-2745.1998.00278.x *
13. Hoyle, G L, Cordiner, H., Good, R. B., & Nicotra, A. B. (2014). Effects of reduced winter duration on seed dormancy and germination in six populations of the alpine herb Aciphyllya glacialis (Apiaceae). Conservation Physiology, 2(1), 1–11. https://doi.org/10.1093/conphys/cou015
14. Hoyle, Gemma L., Venn, S. E., Steadman, K. J., Good, R. B., Mcauliffe, E. J., Williams, E. R., & Nicotra, A. B. (2013). Soil warming increases plant species richness but decreases germination from the alpine soil seed bank. Global Change Biology, 19(5), 1549–1561. https://doi.org/10.1111/gcb.12135
15. Klady, R. A., Henry, G. H. R., & Lemay, V. (2011). Changes in high arctic tundra plant reproduction in response to long-term experimental warming. Global Change Biology, 17(4), 1611–1624. https://doi.org/10.1111/j.1365-2486.2010.02319.x *
16. Kueppers, L. M., Conlisk, E., Castanha, C., Moyes, A. B., Germino, M. J., de Valpine, P., Torn, M. S., & Mitton, J. B. (2017). Warming and provenance limit tree recruitment across and beyond the elevation range of subalpine forest. Global Change Biology, 23(6), 2383–2395. https://doi.org/10.1111/gcb.13561 *
17. Kueppers, L. M., Faist, A., Ferrenberg, S., Castanha, C., Conlisk, E., & Wolf, J. (2017). Lab and field warming similarly advance germination date and limit germination rate for high and low elevation provenances of two widespread subalpine conifers. Forests, 8(11), 1–17. https://doi.org/10.3390/f8110433 *
18. Loranger, H., Zotz, G., & Bader, M. Y. (2016). Early establishment of trees at the alpine treeline: Idiosyncratic species responses to temperature-moisture interactions. AoB PLANTS, 8. https://doi.org/10.1093/aobpla/plw053 *
19. Mallik, A., Wdowiak, J., & Cooper, E. (2011). Growth and reproductive responses of Cassiope tetragona, a circumpolar evergreen shrub, to experimentally delayed snowmelt. Arctic, Antarctic, and Alpine Research, 43(3), 404–409. https://doi.org/10.1657/1938-4246-43.3.404 *
20. Meineri, E., Klanderud, K., Guittar, J., Goldberg, D. E., & Vandvik, V. (2020). Functional traits, not productivity, predict alpine plant community openness to seedling recruitment under climatic warming. Oikos, 129(1), 13–23. https://doi.org/10.1111/oik.06243
21. Meineri, E., Spindelböck, J., & Vandvik, V. (2013). Seedling emergence responds to both seed source and recruitment site climates: A climate change experiment combining transplant and gradient approaches. Plant Ecology, 214(4), 607–619. https://doi.org/10.1007/s11258-013-0193-y
22. Milbau, A., Graae, B. J., Shevtsova, A., & Nijs, I. (2009). Effects of a warmer climate on seed germination in the subarctic. Annals of Botany, 104(2), 287–296. https://doi.org/10.1093/aob/mcp117 *
23. Milbau, A., Vandeplas, N., Kockelbergh, F., & Nijs, I. (2017). Both seed germination and seedling mortality increase with experimental warming and fertilization in a subarctic tundra. AoB PLANTS, 9(5), 1–13. https://doi.org/10.1093/aobpla/plx040 *
24. Mondoni, A., Pedrini, S., Bernareggi, G., Rossi, G., Abeli, T., Probert, R. J., Ghitti, M., Bonomi, C., & Orsenigo, S. (2015). Climate warming could increase recruitment success in glacier foreland plants. Annals of Botany, 116(6), 907–916. https://doi.org/10.1093/aob/mcv101 *
25. Mondoni, A., Rossi, G., Orsenigo, S., & Probert, R. J. (2012). Climate warming could shift the timing of seed germination in alpine plants. Annals of Botany, 110(1), 155–164. https://doi.org/10.1093/aob/mcs097 *
26. Moulton, C., & Gough, L. (2011). Effects of soil nutrient availability on the role of sexual reproduction in an alaskan tundra plant community. Arctic, Antarctic, and Alpine Research, 43(4), 612–620. https://doi.org/10.1657/1938-4246-43.4.612
27. Moyes, A. B., Castanha, C., Germino, M. J., & Kueppers, L. M. (2013). Warming and the dependence of limber pine (Pinus flexilis) establishment on summer soil moisture within and above its current elevation range. Oecologia, 171(1), 271–282. https://doi.org/10.1007/s00442-012-2410-0
28. Munier, A., Hermanutz, L., Jacobs, J. D., & Lewis, K. (2010). The interacting effects of temperature, ground disturbance, and herbivory on seedling establishment: Implications for treeline advance with climate warming. Plant Ecology, 210(1), 19–30. https://doi.org/10.1007/s11258-010-9724-y *
29. Petraglia, A., Carbognani, M., & Tomaselli, M. (2013). Effects of nutrient amendments on modular growth, flowering effort and reproduction of snowbed plants. Plant Ecology and Diversity, 6(3–4), 475–486. https://doi.org/10.1080/17550874.2013.795628
30. Piper, F. I., Fajardo, A., & Cavieres, L. A. (2013). Simulated warming does not impair seedling survival and growth of Nothofagus pumilio in the southern Andes. Perspectives in Plant Ecology, Evolution and Systematics, 15(2), 97–105. https://doi.org/10.1016/j.ppees.2013.02.003
31. Sanhueza, C., Vallejos, V., Cavieres, L. A., Saez, P., Bravo, L. A., & Corcuera, L. J. (2017). Growing temperature affects seed germination of the antarctic plant Colobanthus quitensis (Kunth) Bartl (Caryophyllaceae). Polar Biology, 40(2), 449–455. https://doi.org/10.1007/s00300-016-1972-4 *
32. Shevtsova, A., Graae, B. J., Jochum, T., Milbau, A., Kockelbergh, F., Beyens, L., & Nijs, I. (2009). Critical periods for impact of climate warming on early seedling establishment in subarctic tundra. Global Change Biology, 15(11), 2662–2680. https://doi.org/10.1111/j.1365-2486.2009.01947.x *
33. Shimono, Y., & Kudo, G. (2005). Comparisons of germination traits of alpine plants between fellfield and snowbed habitats. Ecological Research, 20(2), 189–197. https://doi.org/10.1007/s11284-004-0031-8
34. Tercero-Bucardo, N., Kitzberger, T., Veblen, T. T., & Raffaele, E. (2007). A field experiment on climatic and herbivore impacts on post-fire tree regeneration in north-western Patagonia. Journal of Ecology, 95(4), 771–779. https://doi.org/10.1111/j.1365-2745.2007.01249.x
35. Tingstad, L., Olsen, S. L., Klanderud, K., Vandvik, V., & Ohlson, M. (2015). Temperature, precipitation and biotic interactions as determinants of tree seedling recruitment across the tree line ecotone. Oecologia, 179(2), 599–608. https://doi.org/10.1007/s00442-015-3360-0 *
36. Walder, T., & Erschbamer, B. (2015). Temperature and drought drive differences in germination responses between congeneric species along altitudinal gradients. Plant Ecology, 216(9), 1297–1309. https://doi.org/10.1007/s11258-015-0509-1 *
37. Wang, G., Baskin, C. C., Baskin, J. M., Yang, X., Liu, G., Ye, X., Zhang, X., & Huang, Z. (2018). Effects of climate warming and prolonged snow cover on phenology of the early life history stages of four alpine herbs on the southeastern Tibetan Plateau. American Journal of Botany, 105(6), 967–976. https://doi.org/10.1002/ajb2.1104 *
38. Wookey, P. A., Robinson, C. H., Parsons, A. N., Welker, J. M., Press, M. C., Callaghan, T. V, & Lee, J. A. (1995). Environmental Constraints on the Growth, Photosynthesis and Reproductive Development of Dryas octopetala at a High Arctic Polar Semi-Desert, Svalbard. Oecologia, 102(4), 478–489. doi: 10.1007/BF00341360 *
39. Xu, J., Li, W., Zhang, C., Liu, W., & Du, G. (2017). The determinants of seed germination in an alpine/subalpine community on the Eastern Qinghai-Tibetan Plateau. Ecological Engineering, 98, 114–122. https://doi.org/10.1016/j.ecoleng.2016.10.070

**Table S1.** Variables extracted from each article that is part of our systemic review, qualitative analysis and meta-analysis. See Database S1.

| **DatabaseS1 sheet** | **Datum** | **Description** |
| --- | --- | --- |
| Systematic Review | ID | Article unique ID |
|  | AUTHORS | List of the article authors |
|  | YEAR | Year of article publication |
|  | TITLE | Title of the article |
|  | PUBLISHED_IN | Name of journal where the article was published |
|  | PLANT_LIFE_STAGE | Life stage(s) focused on |
|  | STUDY_TYPE | Type of study: observational, in situ experiment, laboratory experiment or a combination |
|  | ENVIRONMENTAL_FACTOR | Environmental factor(s) modified during the study |
|  | CONTINENT | Continent where the study was carried out |
|  | COUNTRY | Country where the study was carried out |
|  | BIOME | Biome where the study was carried out |
|  | PLANT_COMMUNITY | Specific plant community where the study was carried out |
|  | NUMBER_SPECIES | Total number of species investigated in the study |
|  | LIFE_FORM | Life form of the studied species in the study |
|  | DISTRIBUTION | Are the studied species native or exotic to the site where the study was carried out? |
|  | SEEDS_ORIGIN | Origin of the seeds used in the study |
|  | NUMBER_IND_COLLECTED | If seeds were collected by the authors: number of individuals plants that the seeds were collected |
|  | SEEDLINGS_ORIGIN | Origin of the seedlings used in the study |
|  | TRANSPORT_LAB | If it was a lab study: Under what conditions were seeds transported from the field to the lab |
|  | STORAGE_TIME | Storge time after harvest to the beginning of the experiment |
|  | STORAGE_TEMPERATURE | Temperature conditions during the storage time |
|  | STORAGE_LIGHT | Light conditions during the storage time |
|  | PRE-GERMINATION_TREATMENT | If germination was measured during study: what pre-germination treatment were used |
|  | STRATIFICATION_ TEMPERATURE | If cold stratification: under what temperature (°C) |
|  | STRATIFICATION_LIGHT | If cold stratification: under what light conditions |
|  | STRATIFICATION_TIME | If cold stratification: length in weeks |
|  | GERMINATION/SEEDLING_MEDIUM | Identify the growth medium where seed or seedling were established or germinated (e.g. in situ soil, agar, filter paper). |
|  | SUBSTRATE/SOIL TYPE | If field experiment: under what type of substrate/soil type the experiment took place. |
|  | CLIMATE CHANGE EFFECT | What were the changes in the studied environmental factor? |
|  | HOW TREATMENT WAS APPLIED? | How treatment was applied? |
|  | CONTROL | What was the control? |
|  | TREATMENT | What were the treatments? |
|  | LENGTH_MONITORING | For how many weeks was the experiment performed? |
|  | SEED_VIABILITY_TEST | If germination: Did the author perform a viability test before or after the experiment? |
|  | REALISTIC_TREATMENTS? | Does the modification of environmental factors fit the thresholds established by climate change scenarios? |
|  | CITED_PROJECTION | Identify the cited climate change projection during the study |
|  | INCLUDED_QUALITATIVE | Study included qualitative analysis |
|  | INCLUDED_META-ANALYSIS | Study included meta-analysis. |
|  | RECORDED_DATA | If several treatments: which was the control and the used treatment? |
| Qualitative Analysis | ID | Article unique ID |
|  | ENTRY# | Number of entry when multiple entries from the same article are given |
|  | ENVIRONMENTAL_FACTOR | Identify the modified environmental factor(s) |
|  | LIFE_STAGE | Identify the life stage(s) |
|  | SPECIES | Identify target species |
|  | LIFE_FORM | Identify life form of target species |
|  | OVERALL_EFFECT | Identify the overall effect of the environmental factor on the early life history stage, as concluded by the authors: positive, negative, no effect or varies. |
|  | COMMENT | Miscellaneous comments |
| Meta-Analysis | ID | Article unique ID |
|  | ENTRY# | Number of entry when multiple entries from the same article are given |
|  | ENVIRONMENTAL_FACTOR | Modified environmental factor(s) |
|  | LIFE_STAGE | Life stage(s) |
|  | RESPONSE_PARAMETER | Response parameter that was measured in the study |
|  | RESPONSE_UNIT | Unit of response |
|  | SPECIE | Target specie |
|  | LIFE_FORM | Life form of target specie |
|  | MEAN_CONTROL | Mean value of control |
|  | UP_ERROR_CONTROL | When data from graph: Value on y axis of upper error bar for control. n/a=values not extracted from graph |
|  | TYPE_ERROR | Type of error bar given in the study: sd=standard deviation, se=standard error |
|  | SD_CONTROL | Standard deviation of means in control |
|  | N_CONTROL | The sample size for which mean and error for controls are based on |
|  | MEAN_TREATMENT | Mean value of treatment |
|  | UP_ERROR_TREATMENT | When data from graph: Value on y axis of upper error bar for treatment. n/a=values not extracted from graph |
|  | TYPE_ERROR | Type of error bar used in the study: sd=standard deviation, se=standard error |
|  | SD_TREATMENT | Standard deviation of means in treatment |
|  | N_TREATMENT | Sample size for which mean and error for treatment are based on |
|  | COMMENT | Miscellaneous comments |

**Table S2.** Taxonomic identity (family, genus, species) and life form of the taxa studied within the articles that were part of our systematic review. The Study ID is given according to the Database S1.

| **Family** | **Genus** | **Species** | **Life form** | **Study ID** |
| --- | --- | --- | --- | --- |
| Amaranthacea | *Chenopodium* | *aristatum* | Forb | 158 |
|  |  | *foetidum* | Forb | 158 |
|  |  | *glaucum* | Forb | 158 |
|  |  | *hybridum* | Forb | 158 |
|  |  | *iljinii* | Forb | 158 |
|  |  | *prostratum* | Forb | 158 |
|  | *Corispermum* | *tibeticum* | Forb | 158 |
|  | *Kochia* | *scoparia* | Forb | 158 |
| Apiaceae | *Aciphylla* | *glaciaris* | Forb | 246 |
|  | *Bupleurum* | *ranunculoides* | Forb | 826 |
|  | *Tilingia* | *ajanensis* | Forb | 826 |
| Asteraceae | *﻿Achillea* | *millefolium* | Forb | 206 |
|  |  | *moschata* | Forb | 206 |
|  | *Adenostyles* | *leucophylla* | Forb | 296 |
|  | *Ajania* | *salicifolia* | Forb | 158 |
|  |  | *tenuifolia* | Forb | 158 |
|  |  | *unalaschkensis* | Forb | 826 |
|  | *Artemisia* | *desertorum* | Forb | 158 |
|  |  | *edgeworthii* | Forb | 158 |
|  |  | *hedinii* | Forb | 158 |
|  |  | *sieversiana* | Forb | 158 |
|  | *Aster* | *flaccidus* | Forb | 158 |
|  |  | *poliothamnus* | Forb | 158 |
|  |  | *tongolensis* | Forb | 158 |
|  | *Asterolasia* | *trymalioides* | Shrub | 130 |
|  | *Bidens* | *bipinnata* | Forb | 158 |
|  | *Cremanthodium* | *lingulatum* | Forb | 158 |
|  | *Doronicum* | *clusii* | Forb | 296 |
|  | *Erigeron* | *acer* | Forb | 158 |
|  | *Gerbera* | *anandria* | Forb | 158 |
|  | *Gnaphalium* | *supinum* | Forb | 197, 204 |
|  | *Heteropappus* | *crenatifolius* | Forb | 158 |
|  |  | *gouldii* | Forb | 158 |
|  | *Leontodon* | *hispidus* | Forb | 206 |
|  | *Leontopodium* | *haplophylloides* | Forb | 158 |
|  | *Leucanthemopsis* | *alpina* | Forb | 165, 204, 206, 254 |
|  | *Leucanthemum* | *ircutianum* | Forb | 206 |
|  | *Ligularia* | *virgaurea* | Forb | 158 |
|  | *Paraixeris* | *denticulata* | Forb | 158 |
|  | *Saussurea* | *hieracioides* | Forb | 158 |
|  |  | *iodostegia* | Forb | 158 |
|  |  | *salicifolia* | Forb | 158 |
|  | *Scorzoneroides* | *autumnalis* | Forb | 206 |
|  | *Senecio* | *argunensis* | Forb | 158 |
|  | *Serratula* | *strangulata* | Forb | 158 |
|  | *Siegesbeckia* | *pubescens* | Forb | 158 |
|  | *Sinacalia* | *tangutica* | Forb | 158 |
|  | *Solidago* | *virgaurea* | Forb | 154, 349, 826 |
|  | *Taraxacum* | *maurocarpum* | Forb | 158 |
|  |  | *alpina* | Forb | 206 |
|  |  | *ruderalia* | Forb | 206 |
| Betulaceae | *Alnus*  *﻿* | *crispa* | Shrub | 408 |
|  |  | *incana* | Tree | 349 |
|  | *Betula* | *nana* | Shrub | 154, 349 |
|  |  | *papyrifera* | Tree | 408 |
|  |  | *pubescens* | Tree | 349,347 |
| Bignoniaceae | *Incarvillea* | *sinensis* | Forb | 158 |
| Brassicaceae | *Arabis* | *pendula* | Forb | 158 |
|  | *Cardamine* | *alpina* | Forb | 204, 254 |
|  | *Descurainia* | *sophia* | Forb | 158 |
|  | *Draba* | *eriopoda* | Forb | 158 |
|  | *Eruca* | *sativa* | Forb | 158 |
|  | *Lepidium* | *apetalum* | Forb | 158 |
| Campanulaceae | *Adenophora* | *stenanthina* | Forb | 158 |
|  | *Codonopsis* | *nervosa* | Forb | 158 |
|  |  | *pilosula* | Forb | 158 |
|  | *Cyananthus* | *hookeri* | Forb | 158 |
| Caprifoliaceae | *Patrinia* | *sibirica* | Forb | 826 |
| Caryophyllaceae | *Arenaria* | *debilis* | Forb | 158 |
|  |  | *serpyllifolia* | Forb | 158 |
|  | *Cerastium* | *alpinum* | Forb | 350 |
|  |  | *cerastoides* | Forb | 165 |
|  |  | *fontanum* | Forb | 158 |
|  |  | *pedunculatum* | Forb | 197, 296 |
|  | *Colobanthus* | *quitenses* | Forb | 144 |
|  | *Dianthus* | *superbus* | Forb | 158 |
|  | *Lepyrodiclis* | *holosteoides* | Forb | 158 |
|  | *Melandrium* | *apetalum* | Forb | 158 |
|  | *Silene* | *acaulis* | Forb | 206, 347, 349 |
|  |  | *ciliata* | Forb | 220 |
|  |  | *dioica* | Forb | 349 |
|  |  | *vulgaris* | Forb | 206 |
|  | *Stellaria* | *media* | Forb | 158 |
|  |  | *neglecta* | Forb | 158 |
| Crassulaceae | *Rhodiola* | *dumulosa* | Forb | 158 |
|  |  | *kirilowii* | Forb | 158 |
| Cyperaceae | *Carex* | *breviculmis* | Graminoid | 267 |
|  |  | *flavocuspis* | Graminoid | 826 |
|  |  | *pyrenaica* | Graminoid | 826 |
|  |  | *stenantha* | Graminoid | 826 |
|  | *Cyperus* | *flavidus* | Graminoid | 267 |
|  | *Eriophorum* | *angustifolium* | Graminoid | 325 |
|  | *Kobresia* | *graminifolia* | Graminoid | 158 |
|  |  | *kansuensis* | Graminoid | 158 |
| Diapensiaceae | *Diapensia* | *lapponica* | Shrub | 826 |
| Ericaceae | *Bryanthus* | *gmelinii* | Shrub | 826 |
|  | *Cassiope* | *tetragona* | Shrub | 314 |
|  | *Empetrum* | *nigrum* | Shrub | 363 |
|  | *Loiseleuria* | *procumbens* | Shrub | 826 |
|  | *Rhododendron* | *aureum* | Shrub | 826 |
|  | *Therorhodion* | *camtschaticum* | Shrub | 826 |
|  | *Vaccinium* | *myrtillus* | Shrub | 154, 349 |
|  |  | *ovalifolium* | Shrub | 826 |
|  |  | *uliginosum* | Shrub | 349,363, 826 |
|  |  | *vitis-idaea* | Shrub | 347, 349, 826 |
| Euphorbiaceae | *Euphorbia* | *helioscopia* | Forb | 158 |
| Fabaceae | *Astragalus* | *frigidus* | Forb | 349 |
|  |  | *licentianus* | Forb | 158 |
|  |  | *mahoschanicus* | Forb | 158 |
|  |  | *polycladus* | Forb | 158 |
|  |  | *przewalskii* | Forb | 158 |
|  | *Hedysarum* | *tanguticum* | Forb | 158 |
|  | *Lathyrus* | *pratensis* | Forb | 158 |
|  | *Medicago* | *edgeworthii* | Forb | 158 |
|  |  | *lupulina* | Forb | 158 |
|  | *Oxytropis* | *kansuensis* | Forb | 158 |
|  |  | *ochrocephala* | Forb | 158 |
|  | *Phyllodoce* | *aleutica* | Shrub | 826 |
|  | *Thermopsis* | *lanceolala* | Forb | 158 |
|  | *Vicia* | *angustifolia* | Forb | 158 |
|  |  | *cracca* | Forb | 349 |
| Gentianaceae | *Comastoma* | *pulmonarium* | Forb | 158 |
| Gentianaceae | *Gentiana* | *dahurica* | Forb | 158 |
|  |  | *macrophylla* | Forb | 158 |
|  |  | *pseudo-aquatica* | Forb | 158 |
|  |  | *spathulifolia* | Forb | 158 |
|  |  | *straminea* | Forb | 158 |
|  | *Gentianopsis* | *paludosa* | Forb | 158 |
|  | *Halenia* | *elliptica* | Forb | 158 |
|  | *Lomatogonium* | *gamosepalum* | Forb | 158 |
|  | *Swertia* | *erythrosticta* | Forb | 158 |
|  |  | *handeliana* | Forb | 158 |
| Hyoericacea | *Hypericum* | *ascyron* | Forb | 158 |
| Juncaceae | *Juncus* | *thomsonii* | Graminoid | 158 |
|  | *Luzul* | *alpinopilosa* | Graminoid | 197 |
|  |  | *arctuata* | Graminoid | 335 |
|  |  | *multiflora* | Graminoid | 347 |
| Lamiaceae | *Dracocephalu* | *heterophyllum* | Forb | 158 |
|  |  | *tanguticum* | Forb | 158 |
|  | *Elsholtzia* | *densa* | Forb | 158 |
|  | *Lamium* | *amplexicaule* | Forb | 158 |
|  | *Prostanthera* | *cuneata* | Shrub | 130 |
|  | *Salvia* | *roborowskii* | Forb | 158 |
|  | *Schizonepeta* | *tenuifolia* | Forb | 158 |
|  | *Scutellaria* | *amoena* | Forb | 158 |
| Menyanthaceae | *Fauria* | *crista-galli* | Forb | 826 |
| Nothofagaceae | *Nothofagus* | *pumilio* | Tree | 282, 375 |
| Onagraceae | *Epilobium* | *angustifolium* | Forb | 158,347,349 |
|  |  | *palustre* | Forb | 158 |
| Orobanchaceae | *Pedicularis* | *alaschanica* | Forb | 158 |
|  |  | *fletcheri* | Forb | 79 |
|  |  | *polyodonta* | Forb | 158 |
|  |  | *verticillata* | Forb | 158 |
| Papaveraceae | *Meconopsis* | *horridula* | Forb | 158 |
|  |  | *integrifolia* | Forb | 79,158 |
|  |  | *racemosa* | Forb | 79 |
|  | *Papaver* | *radicatum* | Forb | 325 |
| Pinaceae | *Larix* | *decidua* | Tree | 188 |
|  | *Picea* | *abies* | Tree | 188, 205, 408 |
|  |  | *gauca* | Tree | 408 |
|  |  | *jezoensis* | Tree | 61 |
|  |  | *mariana* | Tree | 828 |
|  | *Pinus* | *cembra* | Tree | 188, 827 |
|  |  | *contorta* | Tree | 44 |
|  |  | *engelmannii* | Tree | 118, 135 |
|  |  | *flexis* | Tree | 118, 135, 281 |
|  |  | *sylvestris* | Tree | 154, 205, 347, 349 |
|  |  | *unciata* | Tree | 188, 253, 827 |
|  | *Pseudotsuga* | *menziesii* | Tree | 44 |
| Plantaginaceae | *Pennelianthus* | *frutescens* | Forb | 826 |
|  | *Plantago* | *depressa* | Forb | 158 |
|  |  | *major* | Forb | 158 |
|  | *Veronica* | *alpina* | Forb | 165, 197, 204, 254, 272, 296 |
|  |  | *eriogyne* | Forb | 158 |
|  |  | *stelleri* | Forb | 825 |
| Poaceae | *Agrostis* | *alpina* | Graminoid | 206 |
|  |  | *capillaris* | Graminoid | 206 |
|  |  | *hugoniana* | Graminoid | 158 |
|  | *Anthoxanthum* | *odoratum* | Graminoid | 154 |
|  | *Bromus* | *japonicus* | Graminoid | 158 |
|  |  | *sinensis* | Graminoid | 158 |
|  |  | *tectorum* | Graminoid | 158 |
|  | *Calamagrostis* | *lapponica* | Graminoid | 349 |
|  |  | *purpurea* | Graminoid | 349 |
|  | *Deschampsia* | *flexuosa* | Graminoid | 347, 349 |
|  | *Deyeuxia* | *scabrescens* | Graminoid | 158 |
|  | *Digitaria* | *ciliaris* | Graminoid | 158 |
|  | *Festuca* | *brachyphylla* | Graminoid | 325 |
|  |  | *ovina* | Graminoid | 347, 349 |
|  |  | *rubra* | Graminoid | 158 |
|  | *Poa* | *alpina* | Graminoid | 254 |
|  |  | *annua* | Graminoid | 158 |
|  |  | *laxa* | Graminoid | 197, 296 |
|  |  | *pratensis* | Graminoid | 158 |
|  |  | *tunicata* | Graminoid | 158 |
|  | *Ptilagrostis* | *dichotoma* | Graminoid | 158 |
|  | *Rytidosperma* | *nudiflorum* | Graminoid | 267 |
|  | *Stipa* | *aliena* | Graminoid | 158 |
|  |  | *capillacea* | Graminoid | 158 |
| Polygonaceae | *Bistorta* | *vivipara* | Forb | 335 |
|  | *Oxyria* | *dignya* | Forb | 197, 296 |
|  | *Polygonum* | *macrophyllum* | Forb | 158 |
|  | *Rumex* | *crispus* | Forb | 158 |
| Primulaceae | *Androsace* | *erecta* | Forb | 158 |
|  |  | *gmelinii* | Forb | 158 |
|  |  | *mariae* | Forb | 158 |
|  | *Pomatosace* | *filicula* | Forb | 158 |
|  | *Primula* | *alpicola* | Forb | 79 |
|  |  | *cuneifolia* | Forb | 826 |
|  |  | *orbicularis* | Forb | 158 |
|  |  | *stenocalyx* | Forb | 158 |
| Proteaceae | *Grevillea* | *australis* | Shrub | 130 |
| Ranunculaceae | *Aconitum* | *gymnandrum* | Forb | 158 |
|  | *Anemone* | *rivularis* | Forb | 158 |
|  | *Clematis* | *tangutica* | Forb | 158 |
|  | *Delphinium* | *densiflorum* | Forb | 158 |
|  |  | *grandiflorum* | Forb | 158 |
|  | *Ranunculus* | *tanguticus* | Forb | 158 |
|  | *Thalictrum* | *baicalense* | Forb | 158 |
|  |  | *minus* | Forb | 158 |
|  |  | *uncatum* | Forb | 158 |
| Rosaceae | *Dryas* | *integrifolia* | Forb | 325 |
|  |  | *octopetala* | Shrub | 335,347,349,695 |
|  | *Geum* | *aleppicum* | Forb | 158 |
|  |  | *montanum* | Forb | 206 |
|  |  | *reptans* | Forb | 197, 206, 296 |
|  | *Potentilla* | *bifurca* | Forb | 158 |
|  |  | *longifolia* | Forb | 158 |
|  |  | *matsumurae* | Forb | 826 |
|  |  | *multifida* | Forb | 158 |
|  |  | *potaninii* | Forb | 158 |
|  |  | *supina* | Forb | 158 |
|  |  | *tanacetifolia* | Forb | 158 |
|  | *Sieversia* | *pentapetala* | Shrub | 826 |
|  | *Sorbus* | *aucuparia* | Tree | 188, 827 |
| Rubiaceae | *Galium* | *boreale* | Forb | 158 |
|  |  | *verum* | Forb | 158 |
| Rutaceae | *Phebalium* | *squamulosum* | Shrub | 130 |
| Salicaceae | *Populus* | *balsamifera* | Tree | 408 |
|  |  | *tremuloides* | Tree | 408 |
|  | *Salix* | *artica* | Shrub | 325 |
|  |  | *glauca* | Shrub | 349 |
|  |  | *polaris* | Shrub | 335 |
| Saxifragaceae | *Saxifraga* | *bryoides* | Forb | 197 |
|  |  | *montana* | Forb | 158 |
| Violaceae | *Viola* | *biflora* | Forb | 272 |


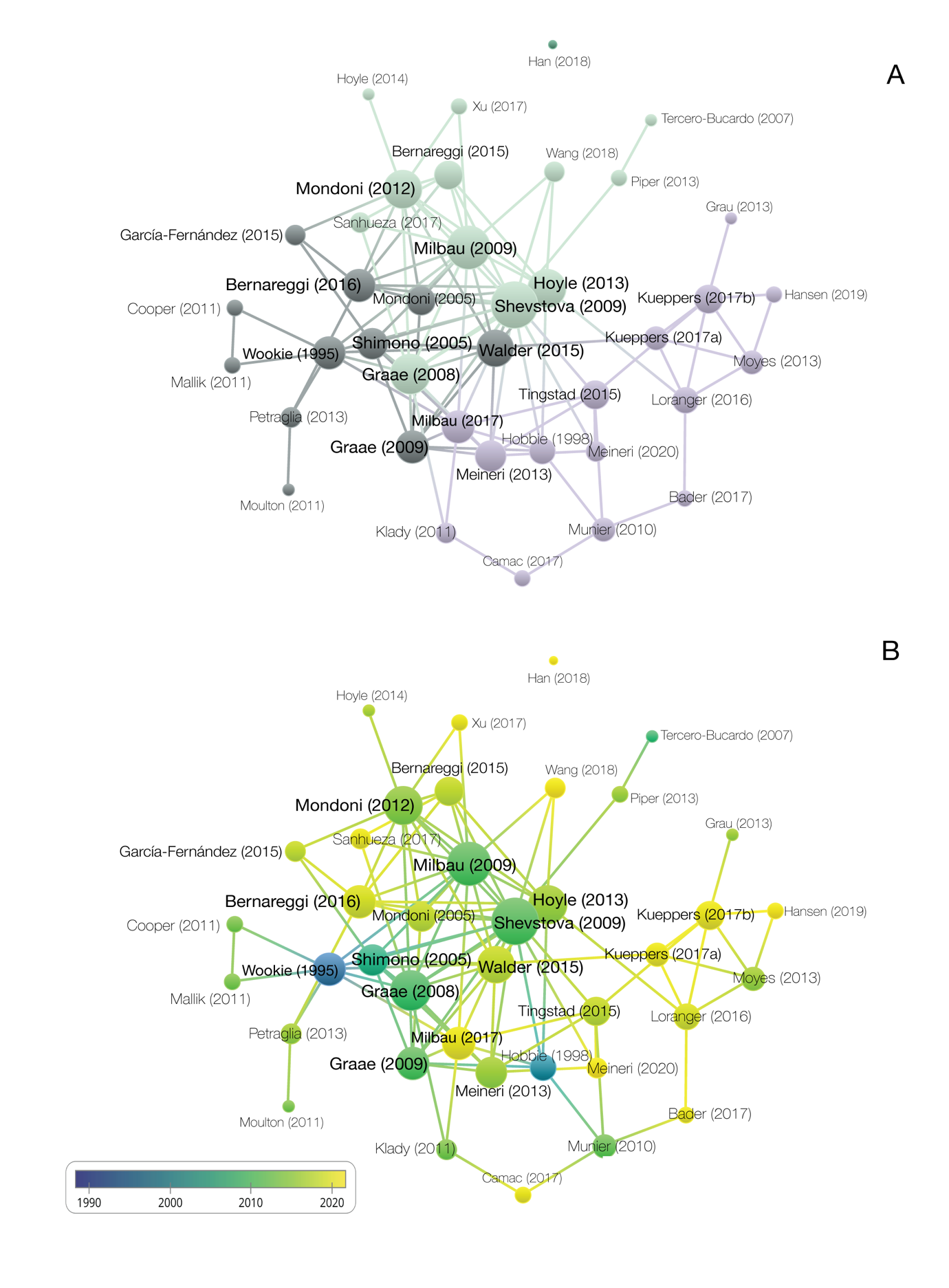


**Figure S1.** Citation map of the articles included in our systematic review (*n* = 39) where the relatedness is determined based on the number of times they cite each other. The size of the nodes is proportional to the number of citations paper have received. (A) Colors show the resulted clusters, (B) Colors show the date of publication.


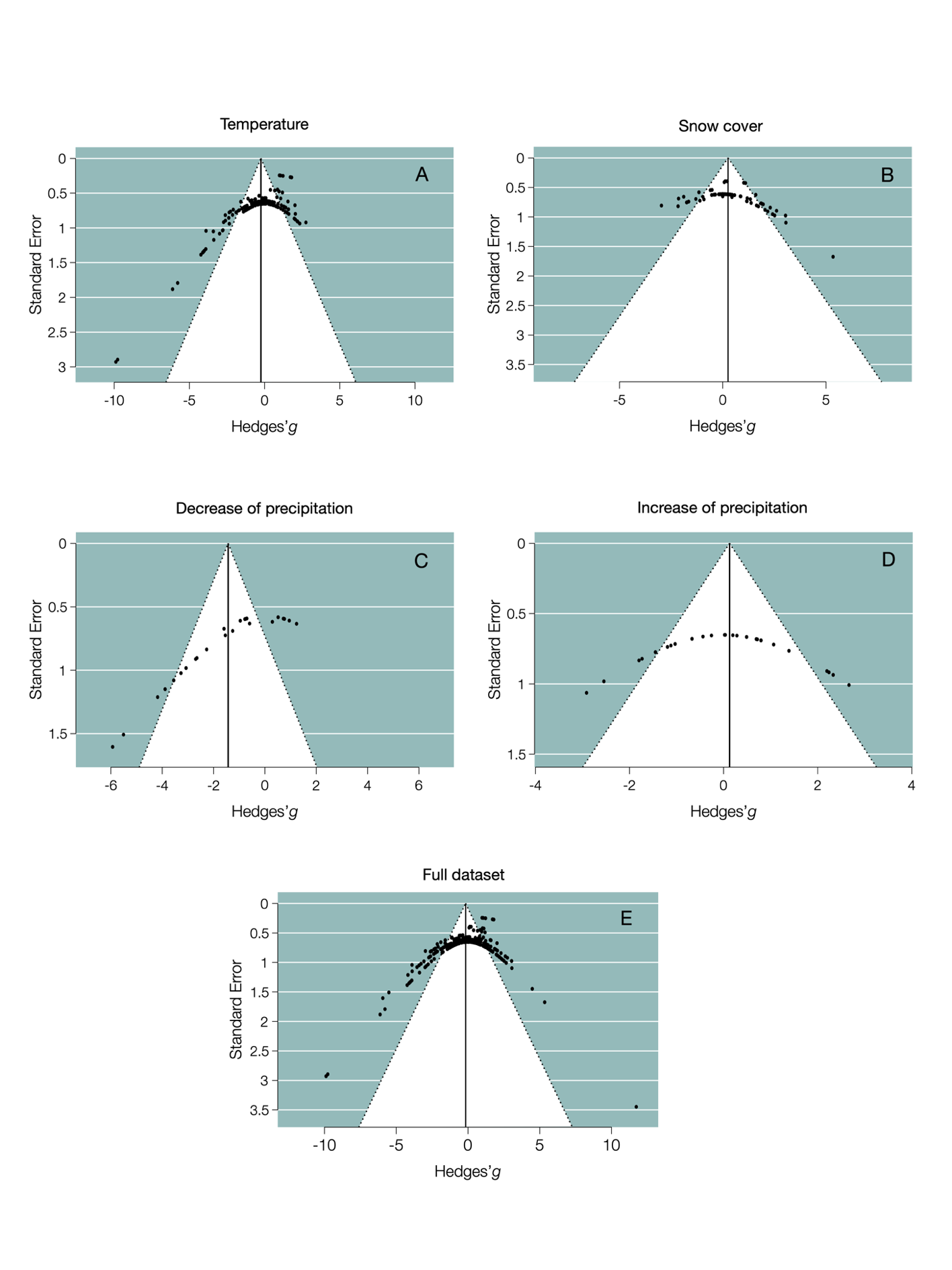
**Figure S2.** Funnel plots for the datasets used in the meta-analysis. The x-axis shows effect size (Hedges’*g*), and the y-axis shows the standard error. The white triangle indicates the region within which 95% of studies are expected to lie in absence of bias and heterogeneity. The black line corresponds to a no intervention effect. Funnel plots are drawn for datasets used to investigate the effect of (A) temperature, (B) snow cover, (C) decrease on precipitation, (D) increase on precipitation and (E) full dataset.

**Checklist S1.** PRISMA (Preferred Reporting Items in Systematic Reviews and Meta-Analyses) checklist [1]

| **Section/topic** | **#** | **Checklist item** | **Reported on page #** |
| --- | --- | --- | --- |
| **TITLE** | | | |
| Title | 1 | Identify the report as a systematic review, meta-analysis, or both. | **1** |
| **ABSTRACT** | | | |
| Structured summary | 2 | Provide a structured summary including (as applicable): background; objectives; data sources; study eligibility criteria, participants, and interventions; study appraisal and synthesis methods; results; limitations; conclusions and implications of key findings; systematic review registration number. | **1** |
| **INTRODUCTION** | | | **2** |
| Rationale | 3 | Describe the rationale for the review in the context of what is already known. | **2** |
| Objectives | 4 | Provide an explicit statement of questions being addressed with reference to participants, interventions, comparisons, outcomes, and study design | **2** |
| **METHODS** | | | |
| Protocol and registration | 5 | Indicate if a review protocol exists, if and where it can be accessed, and, if available, provide registration information including registration number. | **N/A** |
| Eligibility criteria | 6 | Specify study characteristics and report characteristics (e.g., years considered, language, publication status) used as criteria for eligibility, giving rationale. | **3-4** |
| Information sources | 7 | Describe all information sources (e.g., databases with dates of coverage, contact with study authors to identify additional studies) in the search and date last searched. | **2** |
| Search | 8 | Present full electronic search strategy for at least one database, including any limits used, such that it could be repeated. | **2** |
| Study selection | 9 | State the process for selecting studies (i.e., screening, eligibility, included in systematic review, and, if applicable, included in the meta-analysis). | **3-4** |
| Data collection process | 10 | Describe method of data extraction from reports (e.g., piloted forms, independently, in duplicate) and any processes for obtaining and confirming data from investigators. | **3-4** |
| Data items | 11 | List and define all variables for which data were sought and any assumptions and simplifications made. | **Table S1** |
| Risk of bias in individual studies | 12 | Describe methods used for assessing risk of bias of individual studies (including specification of whether this was done at the study or outcome level), and how this information is to be used in any data synthesis. | **4** |
| Summary measures | 13 | State the principal summary measures (e.g., risk ratio, difference in means). | **4** |
| Synthesis of results | 14 | Describe the methods of handling data and combining results of studies, if done, including measures of consistency (e.g., I^2^) for each meta-analysis. | **4** |
| Risk of bias across studies | 15 | Specify any assessment of risk of bias that may affect the cumulative evidence (e.g., publication bias, selective reporting within studies). | **4** |
| Additional analyses | 16 | Describe methods of additional analyses (e.g., sensitivity or subgroup analyses, meta-regression), if done, indicating which were pre-specified. | **N/A** |
| **RESULTS** | |  |  |
| Study selection | 17 | Give numbers of studies screened, assessed for eligibility, and included in the review, with reasons for exclusions at each stage, ideally with a flow diagram. | **5** |
| Study characteristics | 18 | For each study, present characteristics for which data were extracted (e.g., study size, PICOS, follow-up period) and provide the citations. | **Database S1** |
| Risk of bias within studies | 19 | Present data on risk of bias of each study and, if available, any outcome level assessment (see item 12). | **15** |
| Results of individual studies | 20 | For all outcomes considered (benefits or harms), present, for each study: (a) simple summary data for each intervention group (b) effect estimates and confidence intervals, ideally with a forest plot. | **14** |
| Synthesis of results | 21 | Present results of each meta-analysis done, including confidence intervals and measures of consistency. | **8-14** |
| Risk of bias across studies | 22 | Present results of any assessment of risk of bias across studies (see Item 15). | **8** |
| Additional analysis | 23 | Give results of additional analyses, if done (e.g., sensitivity or subgroup analyses, meta-regression [see Item 16]). | **N/A** |
| **DISCUSSION** | |  |  |
| Summary of evidence | 24 | Summarize the main findings including the strength of evidence for each main outcome; consider their relevance to key groups | **12-14** |
| Limitations | 25 | Discuss limitations at study and outcome level (e.g., risk of bias), and at review-level (e.g., incomplete retrieval of identified research, reporting bias). | **13-14** |
| Conclusions | 26 | Provide a general interpretation of the results in the context of other evidence, and implications for future research. | **14** |
| **FUNDING** | |  |  |
| Funding | 27 | Describe sources of funding for the systematic review and other support (e.g., supply of data); role of funders for the systematic review. | **15** |

**References**

1. Liberati, A.; Altman, D.G.; Tetzlaff, J.; Mulrow, C.; Gøtzsche, P.C.; Ioannidis, J.P.A.; Clarke, M.; Devereaux, P.J.; Kleijnen, J.; Moher, D. The PRISMA statement for reporting systematic reviews and meta-analyses of studies that evaluate healthcare interventions: explanation and elaboration. *BMJ* **2009**, *339*, doi:10.1136/bmj.b2700.
